# Serotonergic system modulation holds promise for L-DOPA-induced dyskinesias in hemiparkinsonian rats: A systematic review

**DOI:** 10.17179/excli2020-1024

**Published:** 2020-03-02

**Authors:** Fereshteh Farajdokht, Saeed Sadigh-Eteghad, Alireza Majdi, Fariba Pashazadeh, Seyyed Mehdi Vatandoust, Mojtaba Ziaee, Fatemeh Safari, Pouran Karimi, Javad Mahmoudi

**Affiliations:** 1 Research Center for Evidence-Based Medicine (EBM), Health Management and Safety Promotion Research Institute, Tabriz University of Medical Sciences, Tabriz, Iran; 2 Neurosciences Research Center (NSRC), Tabriz University of Medical Sciences, Tabriz, Iran; 3 Iranian Evidence-Based Medicine (EBM) Center, a Joanna Briggs Institute Affiliated Group; 4 Cardiovascular Research Center, Tabriz University of Medical Sciences, Tabriz, Iran; 5 Phytopharmacology Research Center, Maragheh University of Medical Sciences, Maragheh, Iran; 6 Department of Medical Biotechnology, School of Advanced Medical Sciences and Technologies, Shiraz University of Medical Sciences, Shiraz, Iran

**Keywords:** L-DOPA, levodopa-induced dyskinesias, 6-hydroxydopamine, rat, serotonergic system

## Abstract

The alleged effects of serotonergic agents in alleviating levodopa-induced dyskinesias (LIDs) in parkinsonian patients are debatable. To this end, we systematically reviewed the serotonergic agents used for the treatment of LIDs in a 6-hydroxydopamine model of Parkinson's disease in rats. We searched MEDLINE via PubMed, Embase, Google Scholar, and Proquest for entries no later than March 2018, and restricted the search to publications on serotonergic agents used for the treatment of LIDs in hemiparkinsonian rats. The initial search yielded 447 citations, of which 49 articles and one conference paper met our inclusion criteria. The results revealed ten different categories of serotonergic agents, including but not limited to 5-HT_1A/B_R agonists, 5-HT_2A_R antagonists, selective serotonin reuptake inhibitors (SSRIs), serotonin-norepinephrine reuptake inhibitor (SNRIs), and tricyclic antidepressants (TCAs), all of which improved LIDs without imposing considerable adverse effects. Although there is promising evidence regarding the role of these agents in relieving LIDs in hemiparkinsonian rats, further studies are needed for the enlightenment of hidden aspect of these molecules in terms of mechanisms and outcomes. Given this, improving the quality of the pre-clinical studies and designing appropriate clinical trials will help fill the bench-to-bedside gap.

## Introduction

Levodopa (L-DOPA) is, by far, the most effective therapeutic option for Parkinson's disease (PD). Despite being a well-tolerated medication, L-DOPA induces abnormal involuntary movements (AIMs) in several patients following chronic exposure, commonly known as levodopa-induced dyskinesia (LIDs) (Politis et al., 2014[72]). The mechanisms by which L-DOPA induces motor fluctuations are yet unclear. Nevertheless, some hypotheses have been put forward explaining the phenomenon; such as significant dopamine (DA) swings in the brain caused by a variety of cells (presynaptic hypothesis) along with altered signal-transduction in striatal neurons and abnormal responses in dopaminoceptive neurons (postsynaptic hypothesis) (Cenci, 2014[15]).

Several studies have confirmed the role of serotonergic raphe-striatal neurons in L-DOPA-induced LIDs (Prinz et al., 2013[73]; Tronci and Carta, 2013[79]). Evidence suggests that serotonergic neurons of the central nervous system (CNS) convert orally administered L-DOPA to dopamine (Navailles et al., 2010[61]). These neurons may have a role in the dysregulated metabolism of exogenous L-DOPA and consequently the aberrant striatal release of dopamine in PD patients with LIDs (Kannari et al., 2006[41]; Maeda et al., 2005[49]). Studies performed on rodents and primates have found that modulation of serotonergic activity using 5-HT receptor type 1A and 1B (5-HT1_A _and 5-HT1_B _) agonists such as buspirone and eltoprazine, acute 5-HT transporter (SERT) blockade, or elimination of serotonin (5-HT) afferent terminals may reduce the severity of LIDs without exacerbating PD manifestations (Bezard et al., 2013[5]; Conti et al., 2014[18]; Eskow et al., 2007[26]; Munoz et al., 2008[59]). Although these strategies have yielded promising results in the laboratory, their effects have been barely investigated in the clinic, which might be due to the uncertainty about the impacts of these medications in PD patients. 

Pre-clinical animal studies play a crucial role in the development of novel therapeutics for human via providing evidence for the design of clinical trials. However, we increasingly become aware of the limitations and weaknesses of pre-clinical studies, causing failure during replication of the results from bench to bedside or the “reproducibility crisis” (Begley and Ioannidis, 2015[3]). Systematic reviews of animal studies are detailed, comprehensive, and planned search of the available literature aimed at answering a particular question and reducing bias via detecting and classifying all relevant data. Although systematic reviews of clinical data are extensively available, there exists a paucity regarding the pre-clinical studies. It has been proposed that systematic reviews can increase the so-called “experimental rigor” and the quality of the animal studies and improve translational studies reproducibility (Uman, 2011[82]). This systematic review aimed at finding the old and most up-to-date serotonergic system-based therapeutics for L-DOPA‐induced dyskinesia in hemiparkinsonian rats.

## Methods

### Search strategy

We undertook an electronic search of computer bibliographic databases of MEDLINE via PubMed, Embase, Cochrane, and ProQuest using the keywords “6-hydroxydopamine”, “dyskinesias”, “L- DOPA”, “L-dopa-induced Dyskinesia”, “serotonergic”, “5-HT”, and “serotonine” as follows; (((((((Dyskinesia[Title]) OR Dyskinesia[Title/Abstract])) AND ((((L-dopa[Title/Abstract]) OR L-dopa[Title]) OR Levodopa[Title/Abstract]) OR Levodopa [Title]))) OR ((((((L-dopa induced Dyskinesia[Title/Abstract]) OR L-dopa-induced Dyskinesia[Title/Abstract]) OR Levodopa-induced Dyskinesia[Title/ Abstract]) OR L-dopa induced Dyskinesia [Title]) OR L-dopa-induced Dyskinesia [Title]) OR Levodopa-induced Dyskinesia [Title]))) AND ((((5-HT[Title/Abstract]) OR serotonine[Title/ Abstract]) OR 5-HT[Title]) OR serotonine [Title]). Also, we manually searched the bibliographies of retrieved articles in order not to miss any reports in this regard. Two independent investigators screened the title, abstract, and where necessary, the full texts judging them against the inclusion and exclusion criteria. All disagreements were resolved by the third investigator. Our search was limited to publications in English and other (i.e., non-human) animals. We did not have any time restrictions. 

### Selection criteria

We included all experimental studies that were reported in full-text publications or conference papers and used 6-hydroxydopamine to induce PD, L-DOPA as a treatment for PD, and serotonergic system-based therapeutics for the attenuation of LIDs in rats. The primary outcome measure was to find all serotonergic system-based therapeutics used for LIDs and the secondary outcome measure was to assess their effects in this regard. 

We excluded studies performed on species other than rat (mice and primates) and those that used models other than 6-hydroxydopamine (i.e., MPTP). We also excluded all *ex vivo* or *in vitro* (primary culture or cell line) studies. Further, studies that applied non-serotonergic system-based therapeutics (i.e., glutaminergic, adrenergic, etc.) were excluded.

### Quality assessment

The methodological quality of the included studies was assessed using a modified version of the CAMARADES' study quality checklist. This checklist includes items such as the statement of inclusion and exclusion of animals from the study, blinded assessment of outcome, sample-size calculation, and publication in peer-reviewed journal, randomization to treatment or control, statement of compliance with regulatory requirements, allocation concealment, and statement regarding possible conflict of interest.

## Results

### General study characteristics

**Figure 1:**
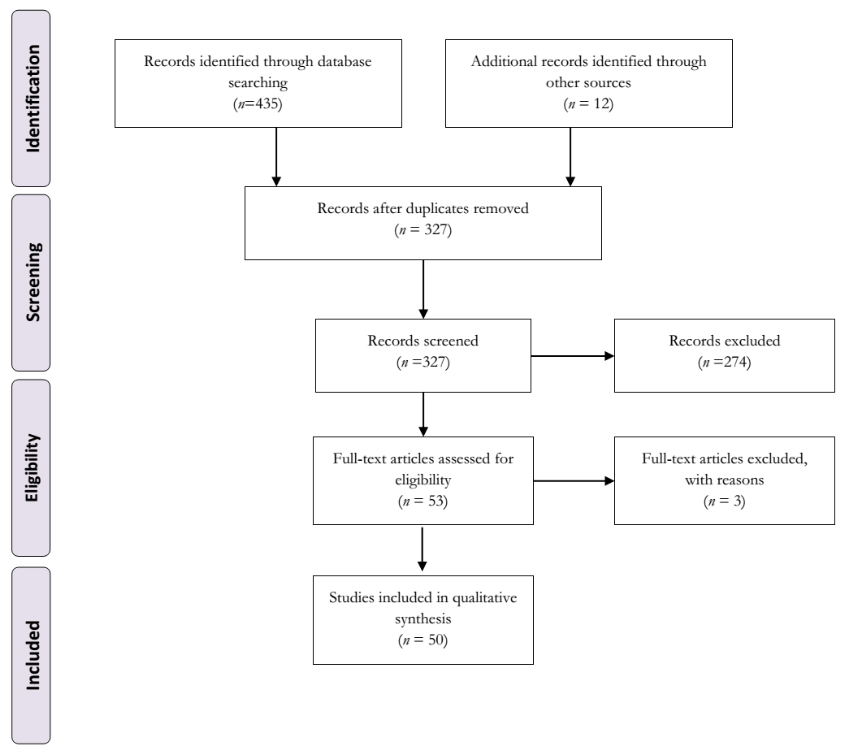
Flowchart of studies selection based on the PRISMA statement for the systematic review.

The search for computer bibliographic databases yielded 447 citations, out of which 49 articles and one conference paper met our inclusion criteria. Figure 1(Fig. 1) shows our search strategy and study selection process. We further divided the included studies into 12 different categories, based on the mechanism of action of the drugs used, as follows; several serotonin 5-HT receptors agonist (n=1), dual D_2_/serotonin 5-HT_1A_R agonist (n=1), dual D1/2 and 5-HT1AR agonist (n=1), 5-HT_1B_R agonist (n=2), mixed 5-HT_1A&B_R agonist (n=11), 5-HT_1A_R agonists including 'biased agonists' and partial agonists (n= 27), serotonin-norepinephrine reuptake inhibitors (SNRIs) (n=1), selective serotonin reuptake inhibitors (SSRIs) (n=7), tricyclic antidepressants (TCAs) (n=1), 5HT_2A/C _and D_2/3_R antagonist (n=1), 5-HT_2A_R antagonists (n=1), and serotonin neuron transplants (n=1). Because some studies tested the effects of more than one serotonergic compound, they were placed in more than one category, and the total number of studies appears to be more than 50. We also found that the most commonly used therapeutic agents were 5-HT_1A_R agonists (n=27) (Table 1(Tab. 1); References in Table 1: Ba et al., 2007[1]; Bezard et al., 2013[4][5]; Bhide et al., 2013[6]; Bibbiani et al., 2001[7]; Bishop et al., 2006[10]; Bishop et al., 2009[9]; Bishop et al., 2012[8]; Carlsson et al., 2007[12]; Carta et al., 2007[13]; Conti et al., 2014[18]; Conti et al., 2016[17]; Dupre et al., 2007[20]; Dupre et al., 2008[21]; Dupre et al., 2011[22]; Dupre et al., 2013[23]; Eskow et al., 2007[26]; Fidalgo et al., 2015[27]; Gerlach et al., 2011[28][29]; Ghiglieri et al., 2016[30]; Iderberg et al., 2013[37]; Iderberg et al., 2015[35][36]; Inden et al., 2012[38]; Jaunarajs et al., 2009[39]; Kuan et al., 2008[44]; Lindenbach et al., 2013[46]; Lindenbach et al., 2015[47]; Marin et al., 2009[52]; McCreary et al., 2016[53]; Meadows et al., 2017[54]; Mo et al., 2010[55]; Munoz et al., 2008[59]; Munoz et al., 2009[58]; Nahimi et al., 2012[60]; Nevalainen et al., 2014[63]; Nishijima et al., 2016[64]; Oh et al., 2002[65]; Paolone et al., 2015[68]; Paquette et al., 2009[69]; Paquette et al., 2012[70]; Pinna et al., 2016[71]; Tani et al., 2010[75]; Taylor et al., 2006[76]; Thomsen and Hansen, 2013[77]; Tomiyama et al., 2005[78]; Tronci et al., 2013[81]; Tronci et al., 2015[80]; Zhao et al., 2014[84]).

### Methodological characteristics 

The methodological features of the included publications were evaluated in 8 different domains according to modified CAMARADES' study quality checklist (see above and Table 2(Tab. 2)). 

According to the nature of this study and its exclusion criteria, all of the included publications were published in peer-reviewed journals (n=49). Less than half of the included studies had performed randomization to treatment or control, and allocation concealment (n=16 and n=21, respectively). However, blind assessment of the outcome was performed in a rather high number of studies (n=31). Only twenty-six studies specified the statement of inclusion and exclusion of animals, and sample-size calculation was performed in none of the included studies. All of the included studies complied with regulatory requirements for animal housing. Also, a low number of these studies (n=20) had the statement of financial disclosure or conflict of interests. In general, the total quality score of the included publications was found to be modest (4.3 out of 8) ranging between 2 and 7.

## Outline of Individual Ttherapeutics

### Several serotonin 5-HT receptors agonist

One publication (a conference paper) investigated the impacts of JM-010, which is a 5-HT_1A_, 5-HT_1D_, 5-HT_1B,_ and 5-HT_1F_Rs agonist, on the severity of LIDs. This study did not specify the dosage and duration of the treatment with JM-010. However, it found that its acute and chronic administration reduced the presentation of AIMs in hemiparkinsonian rats without affecting L-DOPA efficacy and developing tolerance (Thomsen and Hansen, 2013[77]).

### Dual D2/serotonin 5-HT1AR agonists 

Only one publication assessed the impacts of (6aR)-11-amino-N-propyl-noraporphine (SOMCL-171) on LIDs in hemiparkinsonian rats. This medication was found to improve LIDs, including axial, limb and orolingual dyskinesias in a 21-day course of administration without changing the anti-PD efficacy of L-DOPA. This study showed that SOMCL-171 injection to hemiparkinsonian rats activated 5-HT_1A_R and remarkably increased its mRNA expression in the 6-hydroxydopamine-lesioned striatum, both of which contributed to its anti-LIDs effects (Zhao et al., 2014[84]). 

### Dual D1/2 and 5-HT1AR agonist

#### l-Stepholidine (l-SPD)

Mo et al. found that both acute and chronic administration of l-SPD reduced limb and axial dyskinesias in PD rats. Also, the study found that injection of WAY100635 (it should be noted that WAY100635 is not a selective 5-HT_1A_ antagonist so the results as a "proof" of 5-HT_1A_ mechanism should be treated with caution) reversed the therapeutic effects of l-SPD, indicating the key role of 5-HT_1A_R in its antidyskinetic effects. However, it should be noted that WAY100635 is not a selective 5-HT_1A_ antagonist but is also a D_4_ ligand, so all results from studies using this molecule as a "proof" of 5-HT_1A_ mechanism should be treated with caution. Mo et al. also revealed that l-SPD increased the expression level of 5-HT_1A_R mRNA in the lesioned striatum (Mo et al., 2010[55]).

### 5-HT1BR agonists 

We found two articles regarding the effects of CP94253 on LIDs. It was shown that CP94253 administration mitigated LIDs in rats. It was postulated that 5-HT_1B_R activation alleviated LIDs through modulation of the D_1 _receptor function (Jaunarajs et al., 2009[39]). However, Inden et al. (2012[38]) found that the single-dose administration of this agonist had no or little effect in this regard. The difference might result from the route and duration of agonist injection and the fact that Inden et al. assessed the agonist effects on rotational behavior and not dyskinesia.

### Mixed 5-HT1A&B R agonists

#### 5-Hydroxytryptophan (5-HTP)

Our search yielded two citations that used 5-HTP as a treatment for LIDs. Tronci et al. showed that both acute and chronic administration of 5-HTP in hemiparkinsonian rats improved the serotonergic tone and decreased LIDs. This study also showed that various doses of 5-HTP had no difference in their anti-LIDs effect. The authors revealed that 5-HTP-derived serotonin activated both subtypes of 5-HT_1_R which led to displacement of L-DOPA-derived DA to vesicles in the serotonergic neurons and improved LIDs. Also, the stimulation of these receptors decreased glutamate and gamma-Aminobutyric acid (GABA) release in the striatum reducing the severity of LIDs (Tronci et al., 2013[81]). In another study performed by the same authors, the same results were achieved in both acute and chronic treatment groups (Tronci et al., 2015[80]).

#### Eltoprazine

Six citations assessed the effects of eltoprazine on LIDs. All of these studies showed that eltoprazine dose-dependently reduced LIDs, or abnormal involuntary movements (AIMs) both in acute and chronic injections (Bezard et al., 2013[5]; Ghiglieri et al., 2016[30]; Paolone et al., 2015[68]; Pinna et al., 2016[71]; Tronci et al., 2015[80], 2013[81]). However, one study showed that these effects were accompanied by a partial worsening of anti-PD effects of L-DOPA, which might be a concern in this regard (Bezard et al., 2013[5]). Possible suggested mechanisms for anti-LIDs effects of eltoprazine are activation of 5-HT_1A&B_Rs, presynaptic reduction of DA release, decrease in serotonin neurons activity, reestablishment of long-term potentiation (LTP) and synaptic depotentiation in striatonigral medium-sized GABA spiny neurons (the direct pathway) and subsequent regulation of D_1_ receptor-dependent cAMP/ PKA/mGluR1p845 and ERK/mTOR signaling pathways, modulation of NMDA receptor subunits i.e. GluN2A/GluN2B, decrease in the glutamate release induced by activation of 5-HT_1A_R located on the corticostriatal terminals, and decrease in the expression of zif-268 gene (Bezard et al., 2013[5]; Conti et al., 2016[17]; Ghiglieri et al., 2016[30]; Paolone et al., 2015[68]; Pinna et al., 2016[71]; Tronci et al., 2015[80], 2013[81]). Paolone et al., however, argued that 5-HT_1A_Rs-induced decrease in striatal glutamate release and not striatal ectopic dopamine release was apparently responsible for the impacts rendered by eltoprazine (Paolone et al., 2015[68]) This is somehow in contradiction with the findings of other studies mentioned earlier. 

Recent clinical trials assessed the effects of eltoprazine in dyskinetic PD patients yielding promising results in this regard. Svenningsson et al. assessed the effects of a single oral dose of eltoprazine, at 2.5, 5 and 7.5 mg, in 22 patients with PD suffering from LIDs and showed that 5 and 7.5 mg doses were well tolerated and beneficial for this purpose without compromising normal motor responses to L-DOPA. However, no difference was found in Unified Parkinson's Disease Rating Scale (UPDRS) part III scores between the placebo and eltoprazine treatments (Svenningsson et al., 2015[74]). Another clinical trial (please see NCT02439125) evaluated the safety, tolerability, and efficacy of three different doses (2.5, 5 and 7.5 mg) of eltoprazine in treating LID in 60 PD participants; however, its results have not been yet published. 

#### Anpirtoline

We found a citation that assessed anpirtoline impacts on LIDs. Bezard et al. showed that single-dose anpirtoline injection dose-dependently decreased LIDs in hemiparkinsonian rats. Anpirtoline, as opposed to eltoprazine, preferentially activates 5-HT_1B_R subtype, which is located at serotonergic terminals, and at moderate doses shows better results compared with eltoprazine. These effects are at least due to controlled DA, glutamate, and GABA release (Bezard et al., 2013[4]).

#### Combination of ±8-OH-DPAT (1A)/CP-94253 (1B)

Four studies proved the synergic effects of combined administration of ±8-OH-DPAT (1A)/CP-94253 (1B) on the improvement of LIDs in rats. These impacts were shown in both acute and chronic administration of the combination (Carta et al., 2007[13]; Iderberg et al., 2013[37]; Munoz et al., 2009[58], 2008[59]). It has been shown that serotonergic neurons play a crucial role in LIDs; as the existence of serotonin afferent neurons in the nigrostriatal system is crucial for LIDs (Carta et al., 2007[13]). The suggested mechanisms of action of this combination are regulation of DA release from serotonergic neurons, rearrangement of NR2B subunits of NMDA receptors between intra/extra-synaptic parts, and dampening of glutamatergic neurons activity which projects to the striatum from the cortex (Carta et al., 2007[13]; Iderberg et al., 2013[37]; Munoz et al., 2009[58], 2008[59]).

#### ±8-OH-DPAT

By far, ±8-OH-DPAT is the most commonly-used serotonergic drug whose effects on LIDs have been well investigated. We found eleven citations in this regard all of which showed that acute (single dose) or chronic administration of ±8-OH-DPAT, dose-dependently alleviated LIDs in hemiparkinsonian rats (Ba et al., 2007[1]; Bishop et al., 2009[9]; Dupre et al., 2007[20], 2008[21], 2011[22]; Inden et al., 2012[38]; Lindenbach et al., 2013[46], 2015[47]; Nahimi et al., 2012[60]; Tomiyama et al., 2005[78]).

Tomiyama et al. found that chronic administration of ±8-OH-DPAT reduced the number of L-DOPA-induced motor complications, such as rotational behavior. In this study, it was shown that ±8-OH-DPAT blocked the up-regulation of dynorphin (DYN) mRNA in the lesioned striatum. The upregulation of DYN mRNA was associated the motor manifestations of PD, such as dyskinesia. This study also found that activation of 5-HT_1A_R by this agonist decreased the activity of serotonergic neurons in the raphe nucleus and thus modulated L-DOPA metabolism. The same study also showed that ±8-OH-DPAT injection did not change the mRNA levels of the receptor and did not cause receptor down-regulation (Tomiyama et al., 2005[78]). The findings of Ba's study were consistent with the previous study and proved that both acute and chronic ±8-OH-DPAT administration improved L-DOPA-induced motor complications. Ba et al. revealed that treatment with ±8-OH-DPAT decreased the GluR1 hyperphosphorylation leading to a normalized motor response in L-DOPA-treated animal (Ba et al., 2007[1]).

Similarly, Bishop et al. found that the improving effects of ±8-OH-DPAT on AIMs were mediated via 5-HT_1A_R, as the impacts were abolished by antagonization of the receptors using WAY100635. These effects were replicated in both systemic and intrastriatal injection of the agonist and antagonist. This study also found that treatment with ±8-OH-DPAT decreased c-fos expression and changed the transcriptional stimulation of neurons in the postsynaptic striatum. Also, the therapeutic course reduced preprodynorphin (PPD) mRNA in the striatum which was associated with dyskinetic behavior in PD (Bishop et al., 2009[9]). In studies by Dupre et al., it was revealed that ±8-OH-DPAT injection alleviated LIDs by decreasing the release of raphe-striatal DA and corticostriatal glutamate. It also affected postsynaptic factors linked to LIDs such as PPD and glutamic acid decarboxylase (GAD) mRNA (Dupre et al., 2007[20], 2008[21], 2011[22]; Lindenbach et al., 2013[46]). Lindenbach et al. showed that ±8-OH-DPAT treatment bidirectionally modulated pERK expression via stimulation of 5-HT_1A_Rs, decreases D_1_R supersensitivity and thus improved LIDs (Lindenbach et al., 2013[46]). However, these authors showed that 5-HT syndrome was a major concern in this regard and should be taken into account in future studies involving 8-OH-DPAT (Lindenbach et al., 2015[47]). In a positron emission tomography (PET) study by Nahimi et al., it was revealed that ±8-OH-DPAT injection prevented the accumulation of DA in the lesioned striatum and reduced the displacement of DA receptor radioligand and LIDs in L-DOPA-treated rat (Nahimi et al., 2012[60]).

#### BMY-14802

Our search yielded two results that assessed the effects of BMY-14802 on LIDs or AIMs. Both studies showed that single and multiple-dose administration of BMY-14802 in hemiparkinsonian rats reduced the severity of LIDs/AIMs (Bhide et al., 2013[6]; Paquette et al., 2009[69]). Bhide et al. found that BMY-14802 administration dose-dependently decreased orolingual, axial, and limb AIMs, which were abolished upon injection of WAY100635. This suggested the role of 5-HT_1A_R in this regard. Interestingly, this agonist did not change L-DOPA therapeutic efficacy in PD (Bhide et al., 2013[6]). In another study, Paquette et al. achieved the same results and suggested BMY-14802 as a promising candidate for future clinical trials (Paquette et al., 2009[69]). 

#### Sarizotan

It was found that sarizotan dose-dependently reduced AIMs/LIDs in L-DOPA-treated rats (Bibbiani et al., 2001[7]; Gerlach et al., 2011[28]; Marin et al., 2009[52]). Marin et al. found that high-dose (10 ng and 1 µg) injection of sarizotan to subthalamic nucleus significantly reduced the axial, limb, and orolingual dyskinesias score possibly by activation of the 5-HT_1A_R. This study stated that sarizotan had no impact on the pharmacokinetics of L-DOPA. Evidence suggests that sarizotan-mediated stimulation of 5-HT_1A_R dampens the activity of glutamate pathways in the cortex. It also modulates the activity of postsynaptic DA receptors such as D_3_ and decreases the pulsatile activation of DA receptors in the striatum, all of which reduce the severity of L-DOPA-induced AIMs (Bibbiani et al., 2001[7]; Gerlach et al., 2011[28]; Marin et al., 2009[52]). This all can happen in STN as a glutamatergic nucleus of the basal ganglia (Marin et al., 2009[52]).

Clinical trials that have used sarizotan as a treatment for LIDs in PD patients have yielded inconsistent results until now. In a phase II clinical trial (SPLENDID study), Olanow et al. found that treatment with sarizotan (2-10 mg) caused a remarkable decrease in dyskinesia and predominantly its troublesome form. This was manifested by improvements in home diary measures of dyskinesia, UPDRS, and clinical global impression measures determined by both patients and investigators. No change was detected in OFF time in this study. Unfortunately, worsening of Parkinsonism was seen in a number of patients leading to medication discontinuation (Olanow et al., 2004[66]). Afterwards, in a double-blind, randomized, multinational, Phase III study (PADDY-II), the efficacy and safety of sarizotan in PD patients were established (Müller et al., 2006[57]). Accordingly, because sarizotan missed Phase III efficacy endpoints in PADDY-1/2 trials, its further investigation was discontinued. Others found that sarizotan at 2 mg/kg/day dose improved UPDRS and was safe in PD patients with dyskinesia, however, no significant improvements happened compared to placebo on any diary-based measure of dyskinesia or the AIMS score in the sarizotan group (Goetz et al., 2007[31]). 

#### Fenfluramine (FEN) and 3,4-methylenedioxymethamphetamine (MDMA)

In a study by Bishop et al., the effects of FEN and MDMA on AIMs in hemiparkinsonian rats were investigated. Both FEN and MDMA reduced total, axial, and limb AIMs in a dose-dependent manner. However, only MDMA treatment reduced the severity of orolingual dyskinesia. The reported effects were abolished upon administration of 5-HT_1A_R antagonist WAY100635, suggesting a 5-HT_1A_R-mediated mechanism of action. Nonetheless, due to the destructive effects of MDMA on 5-HT nerve fibres and heart valves, its clinical use in humans is unlikely (Bishop et al., 2006[10]).

#### Dextromethorphan

Paquette et al. found that dextromethorphan reduced the expression of L-DOPA-induced AIMs both in Wistar and Sprague-Dawley rats. This effect subsequently reversed by injection of WAY100635, indicating the role of 5-HT_1A_R in this regard. However, because WAY100635 is a non-selective antagonist for -HT_1A_R, this effect might be mediated through the indirect effects of dextromethorphan on these receptors. Besides, dextromethorphan is considered to inhibit serotonin uptake and lead to serotonin overflow, causing activation of 5‐HT_1A_ receptors and reduction of LIDs. As stated by the authors, dextromethorphan may not be the right candidate for suppression of LIDs in PD patients due to its psychogenic and misuse potentials, especially in elderlies (Paquette et al., 2012[70]). 

#### NLX-112 (befiradol or F13640)

Two studies explored the impact of NLX-112 on LIDs in rats. It was found that both acute and chronic administrations of NLX-112 in hemiparkinsonian rats dose-dependently reversed L-DOPA-induced AIMs, including axial, limb, and orolingual dyskinesias (Iderberg et al., 2015[36]; McCreary et al., 2016[53]). Iderberg's study showed that the observed effects disappeared by antagonization of 5-HT_1A_R by WAY100635. This study assumed that the anti-dyskinetic impacts of NLX-112 might be mediated through dampening 5-HT release and the L-DOPA-induced upsurge of DA. However, this study could not prove the effects of NLX-112 on striatal glutamate release (Iderberg et al., 2015[36]). McCreary et al. revealed that a 2-week injection of NLX-112 reversed AIMs proving that there is no desensitization to its anti-dyskinetic impacts. This study confirmed the idea that 5-HT_1A_R modulation decreased the activity of serotonergic neurons and blocked uncontrolled DA release, i.e., “false neurotransmitter.” In line with the previous study, NLX-112 did not affect striatal glutamate release but decreased the L-DOPA-mediated increase in GABA levels in the striatum (McCreary et al., 2016[53]).

#### Buspirone

We found four citations that assessed the effects of buspirone on LIDs/AIMs in PD rats. All of these studies showed that buspirone dose-dependently reduced LIDs/AIMs (Eskow et al., 2007[26]; Gerlach et al., 2011[29]; Lindenbach et al., 2015[47]; Paquette et al., 2009[69]). Lindenbach et al. found that buspirone dose-dependently decreased LIDs; however, its tendency towards causing 5-HT syndrome was lower than that of ±8-OH-DPAT. This study also stated that the frequency of 5-HT syndrome induced by buspirone was higher in rats than humans (Lindenbach et al., 2015[47]). Paquette et al. replicated the same results as the previous study and suggested the key role of 5-HT_1A_R in buspirone-mediated anti-dyskinetic effects (Paquette et al., 2009[69]). Eskow et al. showed that buspirone decreased the severity and development of LIDs and simultaneously enhanced L-DOPA anti-PD efficacy. The authors found that these effects disappeared when 5-HT_1A_R antagonist WAY100635 was used indicating its role in this regard (Eskow et al., 2007[26]). The same findings were reproduced in Gerlach's study (Gerlach et al., 2011[29]).

#### Flibanserin

In a study by Gerlach et al., the effects of flibanserin on LIDs were explored. It was found that flibanserin, as a preferential 5-HT_1A_ receptor agonist, decreased L-DOPA-sensitized controversies circling (i.e., an animal LIDs model) mainly at 10 mg/kg dose in PD rats. The effects of buspirone were found to be superior to those of flibanserin (Gerlach et al., 2011[29]). 

#### F15599

As a G-protein biased agonist, F15599 was applied in 2 citations to alleviate LIDs (Iderberg et al., 2015[35]; Meadows et al., 2017[54]). Meadows et al. found that systemic injection of F15599 significantly decreased AIMs only at higher doses. However, all intra-striatally administered doses of F15599 dose-dependently reduced AIMs. It was suggested that F15599 modulated 5-HT_1A_ hetero-receptors of the forebrain. F15599 renders “biased” stimulation of different subpopulations of 5-HT_1A_ heteroreceptors in the striatum, and thus it is called biased agonist (Meadows et al., 2017[54]). Iderberg et al. found that F15599 at its highest dose decreased total AIMs by 80 % and peak AIMs by 95 %. The study showed that these effects were abolished by antagonization via WAY100635, indicating the role of pre- and post-synaptic cortical 5-HT_1A_ heteroreceptors in this regard. Iderberg et al. also showed that F15599 did not affect the anti-akinetic impacts of L-DOPA. They also found that upon stimulation of 5-HT_1A _the activity of serotonergic neurons and striatal serotonin levels transiently diminished leading to decreased AIMs (Iderberg et al., 2015[35]).

#### F13714

Two citations evaluated the effects of F13714 on LIDs. Meadows et al. showed that both intra-striatal and systemic injection of F13714 dose-dependently reduced LIDs in PD rats. As a biased 5-HT_1A_R agonist, F13714 preferentially activates the pre-synaptic somatodendritic auto-receptors in the dorsal raphe nucleus (DRN). This modulates the ectopic release of false neurotransmitters from the serotonergic neurons of the raphe-striatal system. This study, however, could not exclude the effects of F13714 on other areas of the striatum (Meadows et al., 2017[54]). Iderberg et al. found that L-DOPA-induced AIMs disappeared upon systemic low-dose administration of F13714 which were reversed by WAY100635 injection, indicating the exclusive role of 5-HT_1A_R in this regard. This study also showed that the F13714 administration had no effect on the cortical levels of glutamate, and thus its central mechanism of action depended on the modulation of serotonergic but not the glutamatergic system (Iderberg et al., 2015[35]). 

#### Tandospirone

A study by Iderberg et al. showed that tandospirone abolished LIDs at higher doses, and the impact was blocked by the injection of WAY100635, proving the 5-HT_1A_R-mediated mechanism of action. This study also found that tandospirone did not affect the anti-PD effects of L-DOPA. However, the general anti-LIDs activity of tandospirone was found to be lower than that of F15599 and F13714 due to its partial agonist impacts at 5-HT_1A_R (Iderberg et al., 2015[35]). 

A clinical trial by Kannari et al. showed that administration of tandospirone (15-60 mg/day) in PD patients improved LIDs in more than 50 % of them, but worsened parkinsonian features (Kannari et al., 2002[40]).

#### Piclozan (SUN N4057)

Tani et al. investigated the effects of piclozan on L-DOPA-induced forelimb dyskinesias and showed that piclozan chronic systemic injection significantly reduced their expression. This might be due to a decrease in the excessive L-DOPA-derived DA release from serotonergic neurons of DRN (Tani et al., 2010[75]).

### Serotonin-norepinephrine reuptake inhibitors (SNRI)

#### Duloxetine

Nishijima et al. found that systemic administration of duloxetine significantly enhanced L-DOPA-mediated AIMs. The study showed that duloxetine exacerbated AIMs through inhibition of serotonin transporters. Maintenance of DA in the lesioned striatum was also a mentioned mechanism in this regard (Nishijima et al., 2016[64]).

### Selective serotonin reuptake inhibitors (SSRIs)

#### Fluoxetine

Three studies assessed the effects of fluoxetine on LIDs or L-DOPA-mediated AIMs/rotational behavior (Bishop et al., 2012[8]; Inden et al., 2012[38]; Nevalainen et al., 2014[63]). Inden et al. revealed that acute systemic injection of fluoxetine significantly reduced the expression of rotational behavior, which has the same mechanisms as LIDs. The mentioned effect was abolished by the administration of WAY 100135. Inden et al. argued that anti-LIDs effect of fluoxetine might be due to its indirect effects of 5-HT_1A_R and subsequent efflux of L-DOPA-derived DA.

Further, fluoxetine reversed the L-DOPA-mediated increase in ERK1/2 phosphorylation (Inden et al., 2012[38]). Nevalainen et al. found that fluoxetine co-administration with L-DOPA significantly reduced AIMs score to the baseline in the hemiparkinsonian rats, probably through affecting 5-HT_1A_R. Accordingly, WAY 100635 reversed the impacts of fluoxetine in the same animals. The study postulated that fluoxetine modulated postsynaptic cortical 5-HT_1A_R which controls glutamatergic neurotransmission to the striatum and alleviates LIDs (Nevalainen et al., 2014[63]). Another study by Bishop et al. showed that acute injection of fluoxetine reduced AIMs in L-DOPA-treated hemiparkinsonian rats without affecting its therapeutic efficacy. The authors suggested that SSRIs treatment decreased the turnover of 5-HT and enhanced 5-HT tone proximal to DRN, which led to the activation of regional 5-HT_1A _autoreceptors and subsequent decrease in LIDs (Bishop et al., 2012[8]).

#### Citalopram

The impacts of citalopram on LIDs were assessed across five studies (Bishop et al., 2012[8]; Conti et al., 2014[18]; Fidalgo et al., 2015[27]; Kuan et al., 2008[44]; Lindenbach et al., 2015[47]). Bishop et al. found that citalopram improved LIDs via the mechanisms mentioned under fluoxetine. These effects were dose-dependent and did not interfere with the therapeutic impacts of L-DOPA (Bishop et al., 2012[8]). Dose-dependent reduction of AIMs by injection of citalopram was also seen in Lindenbach's study. This might be due to the activation of 5-HT_1A_R and also 5-HT_1B_R, and an increase in the 5-HT tone. The study also found that citalopram did not cause significant 5-HT syndrome compared to the vehicle group as opposed to buspirone or ±8-OH-DPAT. The authors concluded that potent 5-HT_1A_R agonists such as ±8-OH-DPAT were less favorable in the treatment of LIDs in PD patients as they improved LIDs at the cost of inducing 5-HT syndrome (Lindenbach et al., 2015[47]). Fidalgo et al. demonstrated that both acute and chronic administration of citalopram in hemiparkinsonian rats exerted near-complete suppression of LIDs. It was found that SSRIs including citalopram increased the synaptic concentration of serotonergic neurons and thus stimulated serotonin auto-receptors and modulated LIDs presentation (Fidalgo et al., 2015[27]). In a study by Conti et al. the same results were achieved. This study also found that co-administration of citalopram with L-DOPA prevented the development of LIDs without affecting its therapeutic efficacy. The study proposed that citalopram increased the synaptic levels of 5-HT and indirectly stimulated 5-HT_1A_ somatodendritic autoreceptors in DRN which subsequently regulated striatal DA release and attenuated AIMs. The possible involvement of 5-HT_1B_R cannot be excluded though (Conti et al., 2014[18]). Kuan et al. showed that chronic treatment with citalopram was partially able to abolish LIDs in 6-OHDA-lesioned rats. This was found to be mediated through the complete abolishment of the expression of 5HT_1B_ in the striatum in rats showing LIDs (Kuan et al., 2008[44]).

Although the preclinical studies have been yielded promising results, a clinical trial failed to reproduce the improving effects of citalopram on LIDs in PD patients, which are seen in animal studies (Korsgaard et al., 1986[43]). The translational failure should be addressed in future studies.

#### Paroxetine 

The administration of paroxetine improved LIDs in hemiparkinsonian rats in two studies (Bishop et al., 2012[8]; Conti et al., 2014[18]). Conti et al. found that prolonged injection of paroxetine improved LIDs and prevented their development in rats, and the effects persisted throughout the treatment course. The effects were antagonized by the administration of WAY100635, indicating the 5-HT_1R_R-mediated mechanism of action (Conti et al., 2014[18]). Bishop et al. proved that paroxetine dose-dependently reduced orolingual, axial, and limb AIMs in the rat. This study also found that paroxetine did not change L-DOPA efficacy (Bishop et al., 2012[8]).

A rather recent clinical trial failed to show the improving effects of a 14-day course of paroxetine on LIDs in PD patients, casting doubt on the aforementioned positive effects seen in animal studies (Chung et al., 2005[16]). It is confusing that several positive results have been obtained in the rat but not in the clinic, where SSRIs have been rather ineffective in treating LIDs. As it has been stated by Korsgaard et., it may have some potential explanations; first, the dose used in human studies may not be sufficient to produce anti-LID effects. Second, SSRIs might be able to regulate dopamine levels in the CNS if its turnover is higher than the resting state, and third, SSRIs might play a nonsignificant role in the extrapyramidal syndromes (Korsgaard et al., 1986[43]).

### Tricyclic antidepressants (TCAs)

In a study by Conti et al., the effects of three different TCAs, i.e. desipramine, clomipramine, and amitryptiline on LIDs were investigated. It was revealed that clomipramine dose-dependently decreased L-DOPA-induced AIMs. However, the highest dose of amitriptyline had a non-significant decreasing impact on LIDs. Also, desipramine delayed and did not reduce LIDs in hemiparkinsonian rats without any change in its efficacy. This study concluded that TCAs with the highest affinity for serotonin blockade, i.e., clomipramine are the best options in the treatment of LIDs. The increase in 5-HT tone and subsequent activation of 5-HT_1A_ autoreceptors and normalization of DA release were found to be responsible for the effects seen in this study (Conti et al., 2016[17]).

### 5-HT2A/CR antagonist

#### Quetiapine

A study by Oh et al. found that chronic quetiapine administration reduced LIDs in rats. It was proposed that quetiapine indirectly decreased serotonin release from the presynaptic terminals in the striatum, which led to a decrease in the activation of postsynaptic 5HT_2A/C_R (Oh et al., 2002[65]).

### 5-HT2AR antagonist

#### M100907

Taylor et al. assessed the impacts of M100907 on LIDs in 6-OHDA-lesioned rats. The study found that acute systemic administration of various doses of M100907 reduced the appearance and intensity of D_1_-induced dyskinesia, but not L-DOPA-induced limb, orolingual, or axial dyskinesia in rats. This implies that 5-HT_2A_ antagonism, at least using this medication, may not be a promising strategy, based on rat studies (Taylor et al., 2006[76]). However, later studies on MPTP monkeys using EMD-281014, a potent and highly selective 5-HT_2A_ antagonist, proved that 5-HT_2A_R antagonism might reduce LIDs without compromising the therapeutic effects of L-DOPA (Hamadjida et al., 2018[32]).

### Serotonin neuron transplant

Carlsson et al. showed that serotonin neuron transplant deteriorated LIDs in rats. It was found that in the lesioned striatum DA release from serotonergic neurons terminals as a false neurotransmitter produced swings in the levels of extracellular DA after systemic L-DOPA injection and caused LIDs. This study further confirmed the role of serotonin neurons in the pathophysiology of LIDs (Carlsson et al., 2007[12]). 

## Discussion

### Serotonergic system modulation holds promise for the treatment of LIDs

Oral L-DOPA is the mainstay of treatment for PD patients; however, chronic exposure to the medication results in the development of AIMs in most of these patients, which are known as LIDs. However, the mechanisms are not elucidated (Politis et al., 2014[72]). Evidence suggests that PD patients suffering from LIDs have a more substantial, but a briefer escalation in DA levels in the striatum than those with more stable responses (stable responders) (de la Fuente-Fernandez et al., 2004[19]). 

**Figure 2:**
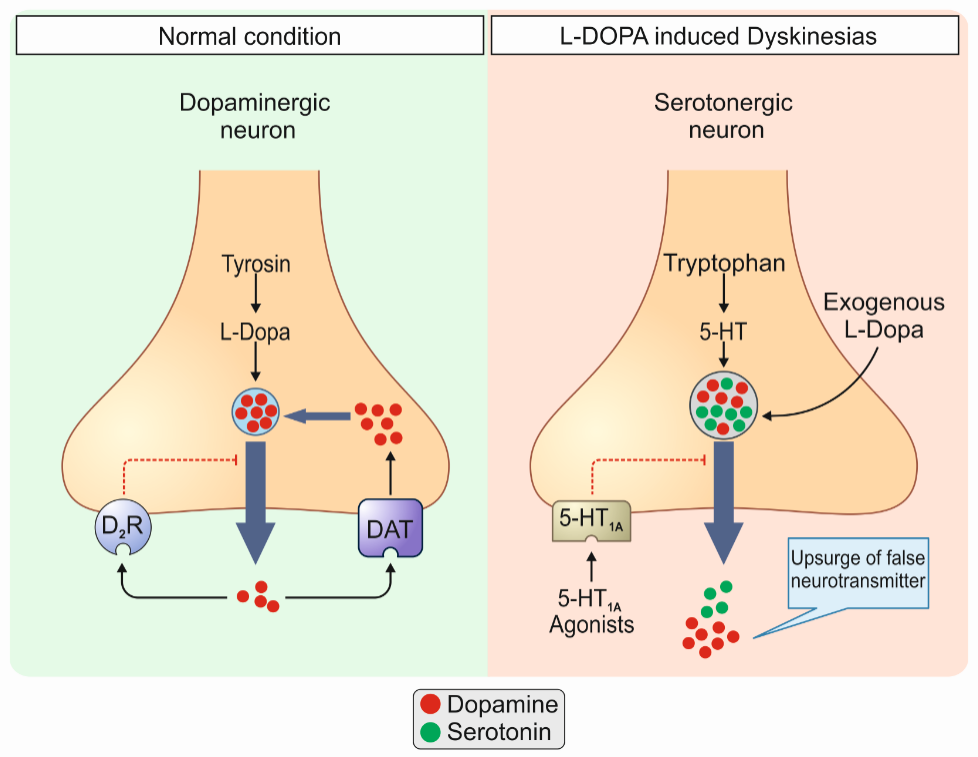
Normal conditions (1) vs. L-DOPA-induced dyskinesias (LIDs) (2). 1: In normal conditions dopaminergic neurons are responsible for the release of dopamine (DA) in the brain. These cells pos-sess autoregulatory mechanisms such as dopamine transporter (DAT) and D2 inhibitory receptors which prevent upsurges of dopamine in the synaptic space. 2: In pathologic conditions i.e., LIDs, serotonergic neurons, which remain intact early in the disease course, take part in the transformation of exogenous L-DOPA to DA, its storage, and release. The produced DA is co-stored with serotonin in serotonergic terminals as a false neurotransmitter and co-released via a process known as compensatory co-transmission. Nonetheless, the lack of autoregulatory mechanisms existing in dopaminergic neurons such as DAT and D2 inhibitory receptors, in serotonergic neurons leads to the aberrant release of DA in the striatum, manifesting as LIDs. D2R, D2 inhibitory receptors; 5-HT, 5-hydroxytryptamine.

Studies have established that the integrity of serotonergic neurons is a critical factor in the development of LIDs, as modulation of serotonergic projections and their activity using serotonergic receptors agonists result in LIDs improvement (Cenci, 2014[15]; Politis et al., 2014[72]). Serotonergic neurons, which remain intact early in the disease course, take part in the transformation of exogenous L-DOPA to DA, its storage, and release (Carta et al., 2007[13]; Tronci and Carta, 2013[79]). The produced DA is co-stored with serotonin in serotonergic terminals as a false neurotransmitter and co-released via a process known as compensatory co-transmission (Mahmoudi et al., 2013[50]). Nonetheless, the lack of autoregulatory mechanisms existing in dopaminergic neurons such as dopamine transporter (DAT) and D_2_ inhibitory receptors in serotonergic neurons leads to the aberrant release of DA in the striatum, manifesting as LIDs (Figure 2(Fig. 2)) (Mahmoudi et al., 2011[51]). 

However, chronic L-DOPA treatment decreases striatal 5-HT nerve fiber density in the absence of dopaminergic neurons (Nevalainen et al., 2014[63]). Indeed, long-term L-DOPA exposure may exert toxic effects (such as the production of 6-OHDA) on serotonergic cells after its uptake, leading to reduction in 5-HT concentration in striatal and extrastriatal areas, further deteriorating dyskinesia in PD patients treated with L-DOPA (Borah and Mohanakumar, 2012[11]; Eskow Jaunarajs et al., 2012[25]). L-DOPA treatment, in the long run, may also increase homocysteine levels causing oxidative stress and excitotoxicity. Activation of these aberrant pathways destructs serotonergic neurons leading to reduced 5-HT levels and 5-HT_1A_R supersensitivity after L-DOPA treatment. Upon a decreased serotonergic activity, dopamine concentration decreases in several brain regions innervated by 5-HT neurons (Lundblad et al., 2009[48]; Müller, 2002[56]; Navailles et al., 2011[62]). All these mechanisms would be finally ensued by the deterioration of LIDs, indicating the vital role of the serotonergic system in the pathogenesis of dyskinesia. 

Mechanisms by which modulation of serotonergic neurons affect LIDs are but are not limited to, regulation of DA release from serotonergic neurons, rearrangement of NR2B subunits of NMDA receptors between intra/extra-synaptic parts, and regulation of the activity of cortical glutamatergic neurons projecting to the striatum (Iderberg et al., 2013[37]; Munoz et al., 2009[58], 2008[59]). 

Also, selective activation of corticostriatal 5-HT_1A_Rs and simultaneously prevention of stimulation of those on thalamocortical and raphestriatal synapses (functional and anatomical selectiveness) result in first, controlled false neurotransmitter (DA) swings in the serotonergic terminals and second, regul-ation of corticostriatal glutamatergic transmission. The reason for the prevention of activation of 5-HT_1A_Rs on thalamocortical and raphestriatal projections resides in the fact that their stimulation may curb the therapeutic efficacy of L-DOPA (Carta et al., 2007[13], 2008[14]; Huot et al., 2011[33]). This, however, should be addressed in future studies.

On the other hand, 5-HT_2A_Rs antagonization produces the same effects described above. Again, to exert anti-dyskinetic effects and simultaneously not compromise L-DOPA efficacy, selective anatomical and functional antagonization seems necessary. This means that the stimulation of those receptors located in corticostriatal areas and the prevention of activation of those reside in nigrostriatal projections (Huot et al., 2011[33]). 

It is worthy to note that modulation of presynaptic 5-HT_1A/1B _Rs also ameliorates D_1 _receptor-induced dyskinesia in animal models of PD by attenuation of striatal glutamate levels. As augmented striatal dopamine D_1_ receptor-induced signaling is involved in LIDS, its decrease invoked by regulation of serotonergic system could be considered a vital therapeutic target for LIDs (Dupre et al., 2013[23]; Jaunarajs et al., 2009[39]).

## Bench to Bedside Translation Failure

Here, it is noteworthy to mention that almost all serotoninergic compounds tested have been under investigation by giant pharmaceutical industries, and because their very first testings in experimental models did not meet the required endpoints, the industry did not support their early clinical testing; as reflected in the few numbers of patients included in these expensive studies (Olivier, 2015[67]).

Several clinical trials have been performed on PD patients, aiming at alleviating LIDs with 5-HT_1A_Rs agonists such as sarizotan. Some of these studies have yielded promising results (Bara-Jimenez et al., 2005[2]; Goetz et al., 2007[31]; Olanow et al., 2004[66]). However, others have failed to reproduce the anti-LIDs impacts of these medications or have shown an improvement of LIDs at the cost of worsening parkinsonism (Chung et al., 2005[16]; Kannari et al., 2002[40]; Korsgaard et al., 1986[43]). This casts doubt over the usefulness of these strategies in PD patients. 

The translational failure seen in clinical trials may stem from several facts as follows; first, as emerges from the results of this study, the quality of the included articles was only modest, highlighting a need for more rigorous design and conducting high-quality experimental studies in this field. It is highly plausible that taking into account measures like randomization, blinding, monitoring physiological factors, included and excluded animals, allocation concealment, and last but not least sample size calculation will reduce the experimental studies bias and fill the gap between bench and bedside. On the other hand, the flaws of clinical trials should not be overlooked. This can be a low statistical power of the trial to identify the real benefit of the treatment under investigation (van der Worp et al., 2010[83]). 

Second, the translational failure may also reside in the fact that the 6-OHDA model of PD in the rat does not entirely mimic the pathophysiology of PD. Acute administration of 6-OHDA to induce hemiparkinsonism in rat imposes substantial denervation of DA neurons, which is highly improbable until the end stages of PD. Also, in the studied animal model, the rodent possesses a rather intact serotonergic system, which is unlikely to be the case in PD patients (Cenci, 2014[15]). Also, dyskinetic animals are reported to have increased 5-HT nerve fiber density, whereas evidence suggests that the 5-HT system becomes degenerated in PD patients. These factors may also affect the outcome of the experimental studies and result in a reproducibility crisis.

Third, different ratios of L-DOPA and benserazide may have been used in combination across various studies; the latter is a decarboxylase inhibitor that does not cross the blood-brain barrier and prevents L-DOPA's peripheral metabolism. This may affect the amount of L-DOPA as well as 3-methoxytyrosine (3-MT) available in the CNS (at the same doses of L-DOPA used) (Kent et al., 1990[42]). Higher levels of 3-MT are associated with higher levels of LIDs, complicating the interpretation of studies results (Lee et al., 2008[45]).

## Concerns over Side Effect Profile

Direct or indirect overactivation of post-synaptic 5-HT_1A_R in rats and 5-HT_1/2A_R in humans or blockade of postsynaptic D_2_ receptors results in a phenomenon which is known as the 5-HT syndrome. Also, the co-administration of L-DOPA with medications that affect the serotonin system imposes a high risk of developing this syndrome. However, this is more like a spectrum as some serotonergic agents pose a higher risk for the syndrome than others (Lindenbach et al., 2015[47]). Unfortunately, due to the similarity of the symptoms between 5-HT syndrome and PD, its diagnosis is occasionally cumbersome leading to diagnoses such as worsening of PD (Ener et al., 2003[24]). The mentioned side effects have been also reported in treatment with the 5-HT_1B/D _agonist, SKF-99101-H, or by the mixed 5-HT_1A_/5-HT_1B_ agonist, 5-HT_2_ antagonist, RU24969 (Paolone et al., 2015[68]).

Another concern over the therapeutic use of serotonergic agents in LIDs is their ability to reduce the efficacy of L-DOPA or exacerbate parkinsonism, as it was seen in eltoprazine administration (Bezard et al., 2013[5]). For instance, Lindenbach et al. showed that 5‐HT_1A_ agonists administration changes spontaneous movement patterns and represses the pro‐motor impacts of L‐DOPA to some extent. Nevertheless, the similarities between 5-HT syndrome and PD worsening symptoms make it difficult to ascribe these features to either one of the phenomena (Lindenbach et al., 2015[47]). So, the use of agents that simultaneously reduce LIDs and maintain L-DOPA efficacy, such as SSRIs, is of high priority. Accordingly, loss of anti-parkinsonian effects has marred the development of 5-HT_1A/1B _agonists, and here again, the rat has been relatively ineffective at uncovering this problem with this category of drugs. This may indicate the narrow therapeutic window of these medications. A new therapeutic avenue has been brought forward by the introduction of compounds with biased agonistic properties, which can dampen LIDs and are simultaneously devoid of anti-PD impacts (Huot et al., 2017[34]).

## Conclusion

LIDs are of the troublesome long-term consequences of treatment with L-DOPA in PD patients. A growing body of evidence shows that the serotonergic system is involved in the pathophysiology of LIDs. This systematic review revealed that serotonergic agents including 5-HT_1A_R agonists, 5-HT_2A_R antagonists, SSRIs, SNRIs, etc. improve LIDs in hemiparkinsonian rats with an almost acceptable side effect profile. However, the possibility of the 5-HT syndrome and worsening parkinsonism should be taken into account. Even though these agents yield promising results in the laboratory, they have not been that much appealing in the clinic. The dramatic effects of these agents observed in animal models and their negligible impacts in clinical trials may be explained by a different role played by the serotonergic system in LIDs in humans compared to other species and, also, the greater intricacy of the human disease as opposed to experimental models. As we discussed earlier, improving the quality of the experimental studies and conducting well-designed clinical trials will help fill the bench-to-bedside gap.

## Conflict of interest

The authors declare that they have no conflict of interest.

## Acknowledgement

This study was supported by a grant from Research Center for Evidence-Based Medicine, Health Management and Safety Promotion Research Institute, Tabriz University of Medical Sciences (Grant no.61131).

## Figures and Tables

**Table 1:**
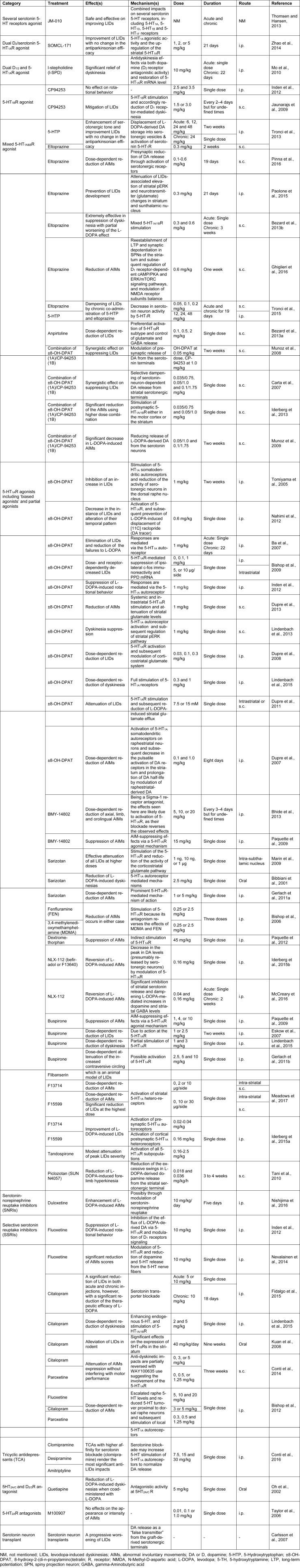
Characteristics of studies investigating the effects of a serotonergic system-based medication on LIDs in 6-hydroxydopamine (6-OHDA) rat model of Parkinson's disease

**Table 2:**
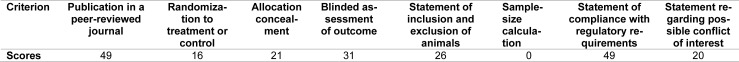
Quality check of the included publications based on modified CAMARADES' animal study quality checklist
